# A line profile‐based double partial fusion method for acquiring planning CT of oversized patients in radiation treatment

**DOI:** 10.1120/jacmp.v13i2.3629

**Published:** 2012-03-08

**Authors:** Huanmei Wu, Qingya Zhao, Minsong Cao, Indra Das

**Affiliations:** ^1^ Purdue School of Engineering and Technology Indiana University Purdue University Indianapolis Indianapolis IN 46202; ^2^ Indiana University Health Proton Therapy Center Bloomington IN 47408; ^3^ Department of Radiation Oncology Indiana University School of Medicine Indianapolis IN 46202 USA

**Keywords:** double scanning, partial registration, oversized patients, planning CT

## Abstract

True 3D CT dataset for treatment planning of an oversized patient is difficult to acquire due to the bore size and field of view (FOV) reconstruction. This project aims to provide a simple approach to reconstruct true CT data for oversize patients using CT scanner with limited FOV by acquiring double partial CT (left and right side) images. An efficient line profile‐based method has been developed to minimize the difference of the CT numbers in the overlapping region between the right and left images and to generate a complete true 3D CT dataset in the natural state. New image processing modules have been developed and integrated to the Insight Segmentation & Registration Toolkit (ITK 3.6) package. For example, different modules for image cropping, line profile generation, line profile matching, and optimized partial image fusion have been developed. The algorithm has been implemented for images containing the bony structure of the spine and tested on 3D CT planning datasets from both phantom and real patients with satisfactory results in both cases. The proposed optimized line profile‐based partial registration method provides a simple and accurate method for acquiring a complete true 3D CT dataset for an oversized patient using CT scanning with small bore size, that can be used for accurate treatment planning.

PACS number: 89

## I. INTRODUCTION

For an effective radiation treatment, it is important to have accurate dose calculation, which depends on CT data and calculation algorithms based on radiological path length of the beam to the isocenter. Situations do arise where beam path cannot be accurately derived due to either lack of CT data or beam passing through materials other than body tissue. In the case of obese patient, scanned on small bore CT scanner or small field of view (FOV) reconstruction, a section of body portion could be truncated during scanning and image reconstruction. Such a scenario produces difficulties in treatment planning process and possibly produces suboptimal beam angles for treatment. In many cases, it requires repeating CT scans, thus causing hardship, delay, extra exposure — possibly without achieving desired CT data.^(^ ^1^ ^,^ ^2^ ^)^ This could be a particularly important issue for particle beam radiotherapy due to the limited range of the beam^(3)^ and advanced treatment techniques like intensity‐modulated radiation treatment (IMRT), where the beam path for each beamlet is unknown or where beam angle optimization (BAO)^(4)^ is used without knowing the beam path.

Thus, accurate 3D CT with complete anatomical information is essential for effective radiation planning and treatment. Evolution in CT imaging has provided wider bore size. For the three major vendors of CT scanners (GE, Siemens, and Philips), the physical bore openings of their largest CT scanners range from 80 cm to 85 cm, while the corresponding reconstruction FOV ranges are between 50 cm and 65 cm. Thus, even if a patient can fit into the scanner, the CT data may have image truncation due to the FOV size. In addition, for some obese patients, even the largest CT scanner is not adequate to fit them suitably. For most medical facilities, due to the viabilities of CT scanners on site, some oversized patients are tightly placed into the CT scanner for 3D CT planning.

**Figure 1:**
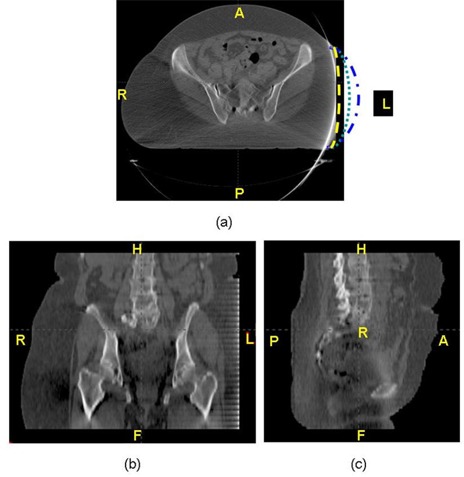
The truncation and deformation of 3D CT planning from different views: (a) the transverse view, which shows the cutoff and deformation of the left side (the dashed lines are the conceptually illustration sample profiles which try to interpolate the truncated region by different imaging processing algorithms); (b) the coronal view, which also shows the truncation on the left side; and (c) the sagittal view of the CT. The CT images were taken by a CT scanner with 45 cm FOV.

Due to limited FOV or bore size, the truncation and deformation are common, very similar to what shown in Fig. 1. For this patient, the right side is in the natural uncompressed (i.e., relaxed and nondistorted) condition. However, the left side has deformation and truncation of anatomical structures. Attempts are made by simply interpolating the missing tissue either manually or mathematically. The dashed lines in Fig. 1(a) are the conceptually illustration sample profiles to interpolate the truncated region by different imaging processing algorithms. Thus, different deformed images can be generated, demonstrated by these different dashed lines that are not true representative of the true CT data.

However, the quality of interpolation does not reflect the actual patient and the CT density within enclosed interpolation is never the same as that of the patient. On the other hand, during treatment delivery, the patients in natural posture are relaxed, nondistorted, and without deformation. Therefore, the CT planning data with limited FOV do not represent the patient anatomy during treatment delivery. Thus, the dose calculation is based on the deformed or truncated planning CT (i.e., the misrepresented anatomy). The anatomical discrepancy on the beam paths results in the difference between the planning dose and the true delivered dose to the patient, particularly to the tumor and other critical structures, which is dependent on the beam energy and the angle of the beam.

A random sampling of the treated patients for lower abdominal sites at our facility showed that about 60% of the CT data are not truly represented, with some degree of truncation and deformation for the planning CT images. The problems of oversized patients with small bore CT scanners are well known in the medical community, but with limited solution. Cone‐beam CT (CBCT) with open gantry geometry integrated with linear accelerator treatment machine has also been proposed. Even though CBCT provides freedom of bore size, FOV image reconstruction is still a major problem. Several investigators have attempted to use CBCT data for treatment planning with limited success.^(^ ^5^ ^,^ ^6^ ^)^ However, the volume truncation issue still exists for onboard CBCT system. Simple image fusion technique, such as taking three images of a patient to reconstruct the 3D image, is recently proposed without accuracy guarantee of image dataset, and with additional exposure to the patient.^(7)^

To overcome truncation and deformation of CT images, several methods have been proposed. One common practice in treatment planning is to avoid beams going through the truncated regions, thus limiting the possible directions of beams, creating a suboptimal treatment plan. The deformed and truncated CT data also create problems in beam angle optimization (BAO) that has been shown to be superior to manual beam selection.^(8)^ When tumor location is known a priori, the patient can be shifted to one side before imaging. This could give a clean image of one side of the patient body, but will result in the deformation and truncation of the other side of patient. When truncated images are present, often simple interpolation of the external contour is carried out which limits the accuracy of dose calculation, since the truncated image is assumed to have CT number of water. The same problems still exist for dose calculation for the truncated and deformed regions. Nonrigid registration approaches for deformation in humans due to respiration phases have been reported.^(8)^ However, the external contour deformation is difficult to resolve using such methods.

The goal of this project is to provide a line profile‐based fusion method for double partial CT data volume registration to accurately acquire true planning CT images of oversized patients using CT scanners with limited bore size and small FOV.

## II. MATERIALS AND METHODS

This section introduces the flow of the double partial CT imaging for oversized patients with small bore scanners and the details of the line profile‐based partial image registration.

### A. Overall introduction of the double partial CT approach

The basic idea of the double partial CT approach is to acquire two 3D CT images, with one focusing on the left and the other focusing on the right side of the patient. There is an overlapped region, which is the region of interest (ROI) between the right portion of the left‐sided CT and the left portion of the right‐sided CT images. The important task is to decide accurately the overlapping region. To achieve this goal, a function on the CT intensity numbers of the ROI is defined as:

(1) $$\Delta I_{\text{ROI}}=\frac{1}{N}\sqrt{{\sum_{V\in ROI}\left[I_{L}(V)-I_{R}(V)\right]}^{2}}$$

where *V* is a voxel in the overlapping ROI, *N* is the total number of voxels, $I_{R}(V)$ and $I_{L}(V)$ are the CT intensity number of the right‐ and left‐sided CT images, respectively.

In our approach, the overlapping ROI is determined by minimizing the difference of the CT numbers between right‐ and left‐sided CT images over the overlapping ROI. To be specific, the objective function is:

(2) $$f=minimize\;\;\;\;\{\Delta I_{\text{ROI}}|(\Delta \text{SI},\Delta \text{AP},\Delta \text{RL})\}$$

where Δ*SI*, Δ*AP*, and Δ*RL* are the overlapping sizes for the superior–inferior (SI), anterior–posterior (AP), and right–left (RL) directions of the ROI, respectively. Upon determination of Δ*SI*, Δ*AP*, and Δ*RL*, the right‐ and left‐sided CT images are combined together to form a full 3D CT Data.

### B. An optimized line profile approach for partial volume registration

To optimize the computational time, a simplified line profile‐based approach is introduced for fast partial volume registration of the two partial CT images, both of which contain the bony structure of the spine. However, the approach can be adapted to other bony structures. The details of the major steps are discussed below.

**Figure 2:**
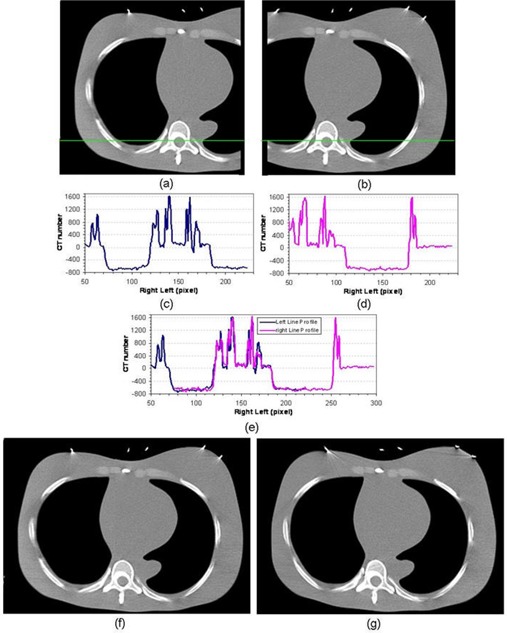
Double partial CT approach on a phantom study: (a) and (b) are one pair of the left and right CT slices; (c) and (d) are the corresponding line profiles for the highlighted horizontal lines; (e) matching of the line profiles; (f) the reconstructed complete CT slice; and (g) the true complete CT slice.

*Step 1 – Double partial CT acquisition* (Fig. 2): Two partial 3D CT images, one for the uncompressed right side and the other for the uncompressed left side, are acquired sequentially by shifting the patient to the left or the right side, with identical imaging parameters, such as kV, mAs, tilts, FOV, slice thickness, and slice width. The same immobilization devices can be applied to minimize patient movement during imaging.

External body markers and cross‐sectioned alignment lasers are used for patient setup. The transverse markers make sure that the two partial CT images have almost the same SI positions and the same number of images. The longitude markers make sure that the two CTs are parallel on the sagittal plane. In addition, as the patient is lying on the CT couch which does not change its height, the coronal planes are kept almost the same for the two images by keeping the table height constant, such that the SI and AP directions have little movement between the two partial CT images. Thus, the ΔSI and ΔAP are very small. The major task is to determine the overlapping along the RL direction.

*Step 2– Spine position identification*: For abdominal and lower abdominal CTs, both partial CT images should contain the bony structure of the spine in a natural state. In our implementation, the spine area is identified using a rectangle, whose size and shape can be customized for each patient to cover the whole spine area in any CT slice. A software module has been developed to automatically identify the spine in the CT images. Domain knowledge, such as the total CT number of the rectangle, the anticipate position of the spine position, and the structure of the spinal cord, has been implemented into the algorithm to avoid false identification of the spine area. For example, one knowledge of the spine area is that the spine should be in the lower part of the transverse view CT image, specifically, lower‐left part of the right partial CT image, and lower‐right part of the left partial CT image.

*Step 3 – Line profile generation*: Without loss of generality, the rectangular center of the left‐sided CT is $\left(x_{c},y_{c},z_{c}\right)$. The CT number of a horizontal line segment of the left‐sided CT image, passing near the middle of the spine, is then extracted, which is the line profile of the left‐sided CT image, as shown in Fig. 2(c), and denoted as:

(3) $$\vec{L}(y_{c},z_{c})=\{I_{L}(y_{c},z_{c},x_{i})|i=0,1,\ldots ,N_{x}\}$$

where $y_{c}$ and $z_{c}$ represent the SI and AP position, and $N_{x}$ is the image width of the x direction (i.e., RL direction). Similarly, the corresponding line profile of the right‐sided CT is extracted, as shown in Fig. 2(d), and denoted as:

(4) $$\vec{R}(y_{c},z_{c})=\{I_{R}(y_{c},z_{c},x_{j})|j=0,1,\ldots ,N_{x}\}$$

*Step 4 – Line profile matching*: The rightmost portion of the left line profile and the leftmost portion of the right line profile are used to identify the overlapping size using pattern recognition based on subsequence matching, including the varied length subsequence matching and the fixed length subsequence matching,^(^ ^9^ ^–^ ^12^ ^)^ as discussed below:

- *Pattern recognition by varied length subsequence matching*: This approach varies the overlapping size Δx, where $\Delta_{\min}\leq \Delta x\leq N_{x}$ $(\Delta_{\min}$ is the minimum overlapping size). The RMS errors of these different overlapping sizes are computed according to Eq. (5) and compared. The real overlapping size Δx will have the minimum RMSE.
  (5) $$\text{RMSE}(\Delta x)=\sqrt{\frac{\sum_{i=0}^{\Delta x}{\left[I_{L}(y_{c},z_{c},x_{N_{x}-i})-I_{R}(y_{c},z_{c},x_{i})\right]}^{2}}{\Delta x}}$$
- *Pattern recognition by fixed length subsequence matching*: Since the overlapping line profile subsequences include the spine width, a subsequence of the left line profile, centered at $\left(x_{c},y_{c},z_{c}\right)$ is chosen *as a query subsequence*. The subsequences with the same length from the right line profile are compared with the query subsequence, and the RMSE between the two subsequences is computed. The corresponding right subsequence with the least RMS error is overlapping with the left query image.

*Step 5 – AP and SI alignment*: The external markers will limit the displacement of the AP and SI direction, but they cannot guarantee 100% accuracy. To address the potential SI shift, the left line profile $\vec{L}(y_{c},\;z_{c})$ is compared with right‐sided CT slices ahead and after the given SI position. For the potential AP shift, $\vec{L}(y_{c},\;z_{c})$ is compared with the horizontal lines of the right‐sided CT above and below the given horizontal line position. Thus, $\vec{L}(y_{c},\;z_{c})$ is compared with $\vec{R}(y_{c-\Delta y},\;z_{c-\Delta z})$. The pair of left and right line profiles with the least RMSE is used to determine shift of the AP and SI directions (i.e., ΔAP and ΔSI).

**Figure 3:**
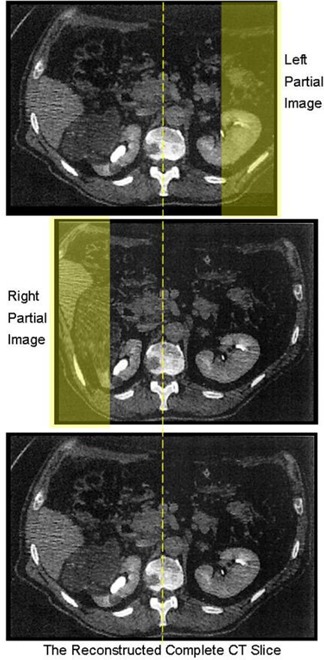
Conceptual illustration of the necessities for the subtraction‐and‐merge due to the deformation of partial images. The shaded areas have deformation and would be cut off before line profile matching and before merging the left and right partial images.

*Step 6 – Partial reconstruction*: If ΔSI≠0, the CT slices are first aligned. The unpaired slices are removed. Thus, the reconstructed 3D CT could be a few slices (usually less than 2) smaller than the original 3D CT. If ΔAP≠0, all the right slices are aligned by moving the voxels up or down. Next, the right‐ and left‐sided CT images are merged to form a complete 3D CT image. Before merging, optimization to exclude the possible deformed regions is performed on both partial CT images. Due to the CT acquisition approach, the rightmost portion of the left‐sided CT and the leftmost portion of the right‐sided CT may be deformed, as the highlighted region shown in Fig. 3. One optimization is to eliminate the possible deformed region from the line profile matching and the reconstruction of the full CT.

In our approach, a subtraction‐and‐merging process is proposed to construct the complete 3D primary planning CT. The basic idea is illustrated in Fig. 3. The far rightmost of the left line profile and the far leftmost of the right line profile can be excluded in the RMSE calculation. Similar optimization can be applied in the partial reconstruction, the deformed rightmost portion of the left‐sided CT and leftmost portion of the right‐sided CT images are subtracted before merging. Regarding the overlapping areas in both partial images, in our implementation, three different ways are proposed to fuse the partial CTs:

- left‐centered merging, in which the overlapping area of the complete 3D CT uses the CT intensity numbers from the left‐sided CT only;
- right‐centered merging, in which the overlapping area of the complete 3D CT uses the CT intensity numbers from the right‐sided CT only;
- average fusion, in which the overlapping area of the complete 3D CT uses the average of the CT intensity numbers from the left‐ and right‐sided CTs.

### C. System implementation

The line profile‐based double CT registration approach is implemented using VC++ with the Insight Segmentation & Registration Toolkit (ITK 3.6).^(13)^ The ITK is a cross‐platform, open‐source application development framework widely used for the development of image segmentation and image registration programs. There are several new modules that have been developed for the project and the major ones are:

- the image cropping module, which generates a new partial 3D image from the given 3D image
- the line profile generation module, which produces the corresponding right and left line profile with the given SI and AP positions
- the line profile matching module, which matches the line profiles of the right and left images, for both variable length and fixed length subsequence matching
- the image fusion module, which merges the two partial images into one complete image for all the three possible fusion approaches

## III. RESULTS

To validate the reliability and performance of the proposed line profile‐based double CT approach, a few validation cases are carried out, including the retrospective analysis of the same CT image, the testing over a human phantom from a Picker UltraZ scanner (GEC, Coventry, UK) having FOV of 45 cm, and the validation of real oversized patient CT images.

### A. Results from the same CT image

The first step in validating the double CT approach is the retrospective analysis of one full CT image. The complete CT image without truncation or deformation of a normal patient is used. The *image cropping module* is used first to generate the left and right partial CTs from the complete 3D CT. In our experiment, the left three‐quarters CT and the right three‐quarters CT images are used for the two partial 3D CT. With the line profile‐based approach, the overlapping size is computed by the minimized RMSE from subsequence matching. The resulting combined CT image is a perfect match of the original complete CT image, which validates the feasibility of the double partial CT approach.

### B. Results from a human phantom

The second step to validate the system is by a human phantom study. The phantom is immobilized on top of the couch of the CT scanner. With the help of laser alignment, two sets of radiopaque markers are put on the surface of the phantom for setup: one set is placed along the lateral direction to approximately align the phantom on the SI direction, and the other set is put along the SI direction to make sure the phantom is parallel on the SI direction during the two imaging.

**Figure 4:**
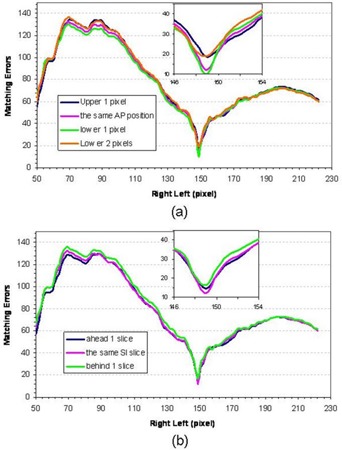
The line profile optimization in locating the partial overlapping areas: (a) the matching RMS error for different slices between the right and left partial images; and (b) the matching errors for different AP positions.

Figure 2(a) and 2(b) show one set of CT slices of the left and right partial images. The corresponding line profiles are shown in Fig. 2(c) and 2(d). The line profile matching module is applied to find the overlapping area. Figure 4(a) displays the matching RMS errors for the same and different slices (different SI positions) between the right and left partial images, which show that the same SI slices yield the smallest RMS error. Thus, there will be no shift among different slices. Similarly, Fig. 4(b) demonstrates the matching errors for different AP positions, which also shows that the RMS error is the smallest when the left image is lower by 1 pixel than the right image. In addition, both figures show that the minimized RMS error is at pixel 149 of the right–left position. Thus, the overlapped area along the RL direction is 149 pixels. Combining all the results, ΔSI=0 slice, ΔRL=149 pixels, and ΔAP=1 pixel. Using these three parameters, the right‐ and left‐sided CT images are merged together using the *CT fusion module* to reconstruct the complete CT. The matched line profile and one reconstructed full CT slice are shown in Figs. 2(e) and 2(f). The reconstructed 3D CT is compared with a ground truth 3D CT, which is a complete 3D CT taken separately of one CT slice (as shown in Fig. 2(g). The two slices match each other well.

### C. Results from the real oversized patient

**Figure 5:**
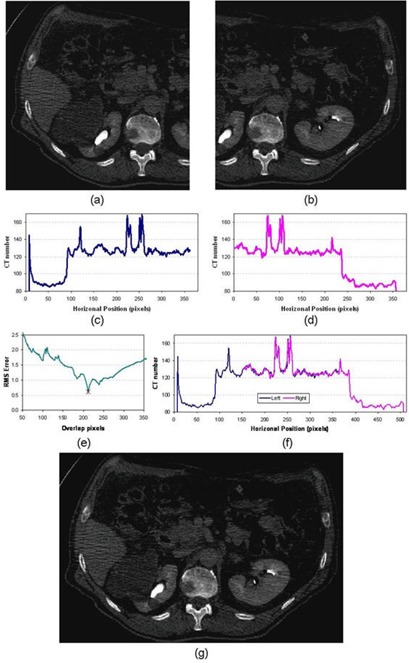
Double partial CT approach on a patient study: (a) left CT slice; (b) the corresponding right slice; (c) the left line profile; (d) the corresponding right line profile; (e) the matching errors; (f) the matching line profiles; and (g) the reconstructed complete CT slice.

The idea and algorithm has been validated for a real patient. The same evaluation process has been performed on the patient as on the phantom. The two partial CT images and sample line profiles are shown in Fig. 5, where the leftmost of the right‐sided CT and rightmost of the left‐sided CT are chopped to avoid deformed area. The line profile matching results for these two line profiles are shown in Fig. 5(e). The minimized RMS error is when the overlapped area includes 212 pixels in the right–left direction. The overlapping left and right line profiles are shown in Fig. 5(f). Additional experiment showed that the SI and AP directions have no shift. Combining the right‐ and left‐sided CT images provides a nondeformed and complete 3D dataset to be used for treatment planning, as shown in Fig. 5(g).

## IV. DISCUSSION

Image fusions have been used for combining various image sets of patients using several mathematical methods successfully.^(^ ^14^ ^–^ ^18^ ^)^ However, such approaches of fusing images will produce deformed images on the lateral side and are not recommended. Rather, the line profile approach and subtraction‐and‐merging processes are needed and which are provided here (discussed in Step 6 of the method above).

Another optimization is to preprocess the partial images before line profile matching. The original images are copied and preprocessed to reduce the noise in profile matching, in which the bone was segmented using a threshold for line profile matching.

The complete reconstructed CT image set has several advantages to be used in the treatment planning system as a primary CT. First, the complete CT data represent the patient in a naturally relaxed state, similar to the treatment condition with minimized deformation. The delivered dose will be almost the same as planned. Second, the full CT has no cutoff regions, which enables the full capability for beam angle optimization.

A potential issue associated with the double CT approach is the extra dose for scanning the patient twice. The CT imaging dose for radiation therapy patients has been a long term debate.^(^ ^1^ ^–^ ^2^ ^)^ The imaging dose in radiation therapy is mostly ignored since the dose is a small fraction of treatment dose. To achieve planning accuracy, two CT images provide a simple and satisfactory solution with slightly extra dose (<5cGy), but much lower than three scans proposed by Fisher et al.^(7)^ This method, however, provides a superior approach for obese and large patients who otherwise cannot be planned accurately.

Another limitation of the proposed approach is that to get the double partial images, a patient must be able to fit into the small CT bore. For extra oversized patients who are not able to fit into the largest CT bore, it is not feasible to use the proposed approach. Instead, an alternative approach should be sought.

## V. CONCLUSIONS

Accurate 3D volume construction for planning is required for effective radiation treatment. However, for small CT scanners and oversized patients, the planning 3D CT may have partial volume truncated or deformed due to the FOV and bore size of the scanner. A double CT approach to accurately acquire planning CT for oversized patients using two partial CT images is introduced. A patient is scanned twice for an uncompressed left‐sided CT and uncompressed partial right‐sided CT. The overlapping regions of the two partial CT images are used to register between the two images. A complete 3D CT plan is reconstructed from the two partial CT images. An optimized and simplified line profile‐based partial registration method has been implemented to provide accurate 3D dataset for partial 3D CT images with bony structure of the spine.

The three validation cases demonstrate the feasibility of the proposed line profile‐based double partial CT approach. This double partial CT approach with line profile is a very useful technique to recover true CT data of an oversized patient from CT scanners with small bore or small FOV.
